# Data on Swiss consumers’ support for different policies aiming to increase sustainability in food consumption and assessment of actor responsibility

**DOI:** 10.1016/j.dib.2025.111570

**Published:** 2025-04-25

**Authors:** Jeanine Ammann, Andreia Arbenz, Gabriele Mack, Michael Siegrist

**Affiliations:** a Agroscope, Research Group Economic Modelling and Policy Analysis, Ettenhausen, Switzerland; b ETH Zurich, Department Health Science and Technology (D-HEST), Consumer Behaviour, Switzerland

**Keywords:** Food policy, Tax, Nudge, Subsidy, Behaviour change, Regulation

## Abstract

We present survey data from 453 Swiss consumers. Data were collected in the German-speaking parts of Switzerland in February 2023 using an online panel provider. The survey included seven distinctive parts. In a first part, personal data including political orientation and consumption behaviour were collected. In a second part, participants assessed the current consumption in Switzerland regarding sustainability. Participants’ food sustainability knowledge was assessed in part three of the survey. In part four, participants rated a total of 19 policy measures for sustainable consumption for their acceptance. Part five dealt with actor responsibility. It included four questions to assess participants’ health consciousness. In part six, we measured participants’ environmental attitudes. In part seven, participants answered questions on who they think was responsible to take action to increase sustainability in consumption and how much trust they had in these actors to do this successfully. The research design was approved by the Ethics Committee of ETH Zurich (approval number: EK 2023-N-04).

Specifications Table

**Table:** 

Subject	Social Sciences.
Specific subject area	Public assessment of policy measures to increase sustainability in food consumption.
Type of data	CSV file (semicolon delimited), SPSS file, survey (PDF) and codebook (PDF).
Data collection	Participants were recruited by LINK (panel provider) and the data were collected through an online survey (accessible from computer and phone) implemented with the survey software Tivian. Data collection took place in the German- speaking parts of Switzerland in February 2023. Quotas were used for age and gender.
Data source location	Institution: Agroscope City/Town/Region: Ettenhausen, Tänikon Country: Switzerland
Data accessibility	**The data is freely available here:** Repository name: Zenodo Data identification number: 10.5281/zenodo.13373845 Direct URL to data:https://zenodo.org/records/13373845
Related research article	Ammann, J., Arbenz, A., Mack, G., & Siegrist, M. (2025). Consumer support of policy measures to increase sustainability in food consumption. Food Policy, 131. https://doi.org/10.1016/j.foodpol.2025.102822 [1].

## 1 Value of the Data

• Data on public support of policy measures is important for policy making.
• Data on predictors of policy support can help identify consumer clusters that are more supportive of certain policy measures.
• The survey contains different methodological approaches which can be used by researchers or teachers.
• This self-reported data can be used to complement studies that used different data collection methods (e.g. voting behaviour, observational studies or choice experiments).

## 2 Background

In a recent review [2], four types of consumer-targeted policy measures (market-based, information-based, regulatory, and nudging) were investigated for their potential to improve sustainable consumption. It has been argued that the implementation of policy instruments, especially in democratic countries, can only succeed if their legitimacy is high [3,4]. With the present dataset, we aimed to build on this evidence and to investigate consumer support of specific policy measures, covering the four types as described in the review. The data presented herein further includes participants’ sustainability assessment of the current consumption behaviour in Switzerland as well as actors’ responsibility to initiate change and trust in them to achieve it.

## 3 Data Description

The data were collected in the German-speaking parts of Switzerland in February 2023. Data were collected through an online survey, which was built using the online survey platform Unipark (Management Questback GmbH, Germany). Participants were recruited through a professional panel provider (LINK, Switzerland). Quotas were used for sex (50 % male and 50 % female) and age (18–35, 36–54, and 55–74 years, 33 % each). A total of 514 participants from the German-speaking parts of Switzerland completed the survey. All participants with an incomplete questionnaire or whose response time was below half of the median response time of the sample (i.e. 328 s) were excluded from the analysis due to data quality concerns. As a result, the final data set consisted of 453 responses. Refer to Table 1 for an overview on the sample. The dataset is freely available from the Zenodo platform [5].

**Table 1:** Sample description (*N* = 453).

		Frequency	%
Sex			
Male		212	46.8
Female		241	53.2
Age	*M = 47.6*	*SD = 15.5*	
Political orientation	*M = 49.8*	*SD = 20.3*	
Education			
No degree or in education		5	1.1
Compulsory school		18	4.0
Vocational apprenticeship/Vocational college/school high		200	44.2
Matura/vocational baccalaureate		48	10.6
Higher technical or vocational training		78	17.2
Technical school or college of education		43	9.5
University/ETH		61	13.5
Place of residence			
Very rural		48	10.6
Rather rural		149	32.9
Sub-urban		129	28.5
Rather urban		73	16.1
Very urban		54	11.9

*Note.* Political orientation was measured on a scale from 0 (left) over 50 (middle) to 100 (right), similar to previous studies [6].

Due to the survey design, participants were required to enter a response in order to proceed with the survey. As a result, there were no missing variables in the dataset. Further, the survey did not include any attention test. The original dataset in wide format (raw; CSV and SPSS file), the survey in German (PDF) and the codebook in English describing the variables (PDF) are freely available online on the Zenodo platform: https://zenodo.org/records/13373845.

## 4 Experimental Design, Materials and Methods

Participants were able to access the survey online or by phone. First of all, participants provided written informed consent. The survey then consisted of seven distinctive parts, which are described in the following (see also Appendix) for the original survey in German and English).

### 4.1 Part 1: Personal information

In this part, individual characteristics including participants gender, age, education level and place of residence were collected. Further, participants indicated how often they consumed meat and dairy on a scale from 1 (multiple times per day) to 6 (never). For easier interpretation, the values were recoded, so that increasing values indicate higher consumption frequencies. As a result, average meat consumption frequency was M = 3.4, SD = 1.1, whereas average dairy consumption frequency was M = 4.1, SD = 1.1. Finally, participants placed themselves on a political left-right scale from 0 (left) over 50 (middle) to 100 (right) using an interactive slider. We included participants’ meat and dairy consumption because previous studies found that consumption can impact individuals’ perception of related policy topics [6,7].

### 4.2 Part 2: Current consumption

In a second part, participants rated the current food consumption in Switzerland on a scale from 1 (our consumption is already sustainable) to 4 (our consumption is not sustainable at all). Again, values were recoded to make it easier to interpret with higher values indicating more sustainability. On average, participants rated the current consumption as rather sustainable (M = 2.5, SD = 0.8).

### 4.3 Part 3: Food sustainability knowledge

In part three, participants’ knowledge on food sustainability was measured using the Food Sustainability Knowledge Questionnaire [8]. The questionnaire contains a total of sixteen multiple choice questions and participants receive one point for each question they answered correctly (see Table 2).

**Table 2:** Participants’ food sustainability knowledge based on the FSKQ (*N* = 453).

	Frequency	Percentage
Correct answers on the FSKQ		
0	8	1.8
1	12	2.6
2	13	2.9
3	22	4.9
4	18	4.0
5	18	4.0
6	34	7.5
7	41	9.1
8	32	7.1
9	45	9.9
10	55	12.1
11	61	13.5
12	32	7.1
13	28	6.2
14	19	4.2
15	13	2.9
16	2	0.4

### 4.4 Part 4: Policy measures for sustainable consumption

In the fourth part of the survey, participants were informed that the food system accounts for a significant amount of greenhouse gas emissions and that different strategies exist to reduce diet-related greenhouse gas emissions. After reading this introduction, they were presented with 19 policy measures and asked to indicate for each of them how much they would support it on a 7-point Likert scale ranging from 1 (I do not think it is good at all) to 7 (I think it is very good). The phrasing of the question and some items were similar to previous research investigating government intervention for a healthier diet [7]. The endpoints and the middle of the response scale were identified through verbal description.

This part of the survey builds on the results from a recent review [2]. Policy measures were designed to cover the four product categories meat, vegetables, dairy and not specified. Further, it was aimed to cover five types of policy measures in accordance with Ammann, Arbenz [2], that is, information, nudge, subsidy, tax and regulatory. Item creation relied on previous research [7,9, 10]. However, it was not possible to come up with a realistic item in the unspecific and regulatory combination, which is why the total amount of items created was 19 (see Table 3).

**Table 3:** List of all 19 policy measures included in the study, their item number and food category targeted.

Policy measure	Product Group	#	English translation
Information	Meat	1^a^	Greater emphasis on meatless recipes in cooking school.
	Dairy	2^b^	Information campaigns informing about the negative environmental impact of dairy products.
	Vegetable	3^b^	In the supermarket, seasonal vegetables are marked with a label.
	Non-Specific	4^b^	At the point of sale, information on the environmental impact of all food products must be provided (e.g. with a label).

Nudge	Meat	5^c^	In canteens, the first named dish must always be meat-free.
	Dairy	6^d^	In the store, shelves with plant-based milk alternatives are clearly marked (e.g. with a large green sign).
	Vegetable	7^d^	In canteens, at least one dish on the menu must include seasonal vegetables.
	Non-Specific	8^d^	In canteens, smaller portions are served with the possibility of a second helping.

Tax	Meat	9^d^	Tax on meat products to reduce sales
	Dairy	10^d^	Tax on dairy products to reduce sales
	Vegetable	11^a^	Tax on non-seasonal vegetables (e.g. zucchini, peppers or eggplants in winter) to reduce sales
	non-Specific	12^a^	Tax on foods that are harmful to the environment

Subvention	Meat	13^a^	Meat alternatives are subsidized to be cheaper than meat.
	Dairy	14^a^	Dairy alternatives are subsidized to be cheaper than milk.
	Vegetable	15^a^	Seasonal vegetables (e.g. squash, leeks, cabbage in winter) are subsidized to promote sales.
	Non-specific	16^a^	Subsidies on environmentally friendly food

Regulation	Meat	17^d^	Canteens must offer exclusively meatless dishes two days per week.
	Dairy	18^b^	Advertising ban on dairy products
	Vegetable	19^d^	No vegetables imported by airplane may be offered in stores.

Note: Support was measured on a scale from 1 (I do not think it is good at all) to 7 (I think it is very good). Items were based on:

a authors

b [7].

c [10].

d [9].

### 4.5 Part 5: Health consciousness

In part five of the survey, participants completed the four items from the health consciousness scale by Dohle, Hartmann and Keller [11]. All items were rated on a 7-point Likert scale ranging from 1 (do not agree at all) to 7 (totally agree). Sample items were “I think it is important to eat healthily” and “My health is dependent on how and what I eat.” The scale’s reliability was good (4 items, Cronbach’s α =.75, M = 5.1, SD = 1.1). We included health consciousness to check for synergies between sustainability and health perception, which was found in previous research [12,13].

### 4.6 Part 6: Environmental attitude

In part six of the survey, participants’ environmental attitudes were assessed using the New Ecological Paradigm scale [NEP, 14] . The scale contains 15 items, which participants were asked to evaluate on a 5-point Likert scale ranging from 1 (do not agree at all) to 5 (totally agree). Sample items are: “Humans have the right to modify the natural environment to suit their needs” and “Human ingenuity will ensure that we do not make the Earth unliveable”. The scale’s reliability was good (15 items, Cronbach’s α =.78, M = 3.6, SD = 0.5).

### 4.7 Part 7: Responsibility

In part seven, participants were asked to rate the four actors (agriculture, retail, consumers and government / politics) each on a scale from 1 (no responsibility at all) to 7 (a great amount of responsibility), indicating how much they believed each actor was responsible for a sustainable food consumption. In a second step, participants indicated for each actor how confident they were that they will promote sustainable food consumption on a scale from 1 (not confident at all) to 7 (very confident) see Table 4).

**Table 4:** Participants’ food sustainability knowledge based on the FSKQ (*N* = 453).

	Responsibility		Confidence	
	M	SD	M	SD
Agriculture	5.6	1.3	4.3	1.6
Retail	5.5	1.3	4.0	1.5
Consumers	5.8	1.3	4.1	1.6
Government/politics	5.4	1.5	3.9	1.6

## Limitations

When working with this data, researchers need to keep in mind that it was obtained from the German-speaking parts of Switzerland.

## Ethics Statement

All participants involved in the study provided their written, informed consent to participate. Participation was voluntary and could be withdrawn at any time. Participants remained anonymous and their responses were dealt with in confidence. Ethical approval was obtained from the Ethics Committee of ETH Zurich (approval number: EK 2023-N-04).

## CRediT authorship contribution statement

**Jeanine Ammann:** Writing – review & editing, Writing – original draft, Supervision, Project administration, Methodology, Formal analysis, Data curation, Conceptualization. **Andreia Arbenz:** Writing – review & editing, Methodology, Investigation, Formal analysis, Data curation, Conceptualization. **Gabriele Mack:** Writing – review & editing, Resources. **Michael Siegrist:** Writing – review & editing, Resources, Project administration, Conceptualization.

## Data Availability

ZenodoDataset on consumer acceptance of policy measures for sustainable food consumption (Original data).

## Appendix Supplementary materials

Image, application 1
