# Supplementary material for: Data on Swiss consumers’ support for different policies aiming to increase sustainability in food consumption and assessment of actor responsibility
Source: Data Brief. 2025 Apr 25;60:111570. doi: 10.1016/j.dib.2025.111570 (PMC12104707; doi:10.1016/j.dib.2025.111570)
Supplement: Supplementary file 1 [file mmc1.docx]

# Appendix

Table A1: Original survey and English translation

| **German (original)** | **English (translation)** |
| --- | --- |
| **Begrüssung / Einverständnis** | **Welcome / Consent** |
| Einverständnis  [1] Ich stimme zu und möchte an der Studie teilnehmen.  [2] Ich stimme nicht zu und möchte nicht an der Studie teilnehmen. | Consent  [1] I agree and would like to participate in the study.  [2] I do not agree and do not wish to participate in the study. |
| **1.1 Geschlecht** | **1.1 Gender** |
| Teil 1: Angaben zu Ihrer Person  Wir bitten Sie nun einige Angaben zu Ihrer Person zu machen. Bitte geben Sie uns Ihr Geschlecht an.  *Geschlecht*  [1] Mann  [2] Frau  [3] anderes  [4] möchte nicht antworten | Part 1: Personal information  We would now like to ask you to provide some personal information. Please tell us your gender.  *Gender*  [1] man  [2] woman  [3] other  [4] do not wish to answer |
| **1.2 Jahrgang** | **1.2 Year of Birth** |
| Bitte geben Sie uns Ihr Geburtsjahr im Format JJJJ an.  *Geburtsjahr*  [ _ _ _ _ ] | Please enter your year of birth in the format YYYY.  *Year of birth*  [ _ _ _ _ ] |
| **1.3 Ausbildung** | **1.3 Education** |
| Kreuzen Sie bitte Ihre höchste, abgeschlossene Ausbildung an.  *Ausbildung*  [1] Kein Abschluss / In Ausbildung  [2] Obligatorische Schule  [3] Berufslehre / Berufsfachschule /Handels(mittel)schule  [4] Maturität / Berufsmaturität  [5] Höhere Fach- oder Berufsausbildung  [6] Fachhochschule oder pädagogische Hochschule  [7] Universität / ETH | Please indicate the highest level of education you have completed.  *Education*  [1] no qualification / in training  [2] compulsory school  [3] vocational apprenticeship / vocational school / trade (secondary) school  [4] Matura / vocational baccalaureate  [5] higher specialised or vocational training  [6] university of applied sciences or teacher training college  [7] University / ETH |

| **1.4 Wohngegend** | **1.4 Place of Residence** |
| --- | --- |
| Was trifft am ehesten auf aktuelle Wohngegend zu?  [1] sehr ländlich  [2] eher ländlich  [3] vorstädtisch  [4] eher städtisch  [5] sehr städtisch | What describes your current neighbourhood most accurately?  [1] very rural  [2] rather rural  [3] suburban  [4] rather urban  [5] very urban |
| **1.6 Essenspräferenz** | **1.6. Food Preference** |
| Ernährung  Wie oft essen Sie Fleisch?  [1] mehrmals täglich  [2] täglich  [3] 4-6 mal pro Woche  [4] 1-3 mal pro Woche  [5] selten  [6] gar nie | Nutrition  How often do you eat meat?  [1] several times a day  [2] daily  [3] 4-6 times a week  [4] 1-3 times a week  [5] rarely  [6] never at all |
| **Essenspräferenz_Milch** | **Food Preference Milk** |
| Ernährung  Wie oft essen Sie Milchprodukte?  [1] mehrmals täglich  [2] täglich  [3] 4-6 mal pro Woche  [4] 1-3 mal pro Woche  [5] selten  [6] gar nie | Nutrition  How often do you eat dairy products?  [1] several times a day  [2] daily  [3] 4-6 times a week  [4] 1-3 times a week  [5] rarely  [6] never at all |

| **1.7 Politische Orientierung** | **1.7 Political Orientation** |
| --- | --- |
| Wo ordnen Sie sich auf einer politischen links-rechts Skala ein?  Bitte klicken Sie in den Balken, um Ihre Antwort abzugeben.  (interactive slider scale: 0 (Links) – 50 (Mitte) – 100 (Rechts) | Where do you position yourself on a political left-right scale?  Please click in the bar to give your answer.  (interactive slider scale: 0 (Left) – 50 (Neutral) – 100 (Right) |
| **2. Schweizerischen Lebensmittelkonsum** | **2. Swiss Food Consumption** |
| Zu Beginn möchten wir von Ihnen erfahren, wie Sie den schweizerischen Lebensmittelkonsum aus nachhaltiger Sicht bewerten.  Bitte wählen Sie eine der folgenden Aussagen aus.  [1] Unser Konsum ist bereits nachhaltig.  [2] Unser Konsum ist fast nachhaltig.  [3] Unser Konsum ist eher nicht nachhaltig.  [4] Unser Konsum ist gar nicht nachhaltig. | To begin with, we would like to find out how you rate Swiss food consumption in terms of sustainability.  Please select one of the following statements.  [1] Our consumption is already sustainable.  [2] Our consumption is almost sustainable.  [3] Our consumption is rather unsustainable.  [4] Our consumption is not sustainable at all. |
| **3. Ernährungsbezogenes Umweltwissen in Anlehnung an (Hartmann et al., 2021)** | **3. Nutrition-Related Environmental Knowledge Based on (Hartmann et al., 2021)** |
| Die grösste Umweltbelastung im Lebensmittelbereich entsteht bei …  Bitte wählen sie eine zutreffende Antwort.  *Q1*  [1] der Produktion  [2] der Lagerung  [3] der Verpackung  [4] dem Transport (Schiff & LKW)  [5] Weiss nicht | The greatest environmental impact in the food sector is caused by ...  Please select an appropriate answer.  *Q1*  [1] production  [2] storage  [3] packaging  [4] transport (ship & lorry)  [5] do not know |

| Die Produktion von Fleisch und Milchprodukten führen zu mehr CO2 Emissionen pro kg als die Produktion von Gemüse?  Bitte wählen Sie eine zutreffende Antwort.  *Q2*  [1] Richtig  [2] Falsch  [3] Weiss nicht | The production of meat and dairy products leads to more CO2 emissions per kg than the production of vegetables?  Please select an appropriate answer.  *Q2*  [1] correct  [2] incorrect  [3] do not know |
| --- | --- |
| Die Produktion von 1 kg Rindfleisch verursacht mehr Treibhausgase als die Produktion von 1 kg Weizen.  Bitte wählen Sie eine zutreffende Antwort.  *Q3*  [1] Richtig  [2] Falsch  [3] Weiss nicht | The production of 1 kg of beef causes more greenhouse gases than the production of 1 kg of wheat.  Please choose an appropriate answer.  *Q3*  [1] correct  [2] incorrect  [3] do not know |
| Die negativen Effekte des Lebensmittelkonsums auf die Umwelt können durch die Umstellung auf eine vegetarische Ernährung (kein Konsum von Fleisch) reduziert werden.  Bitte wählen Sie eine zutreffende Antwort.  *Q4*  [1] Richtig  [2] Falsch  [3] Weiss nicht | The negative impacts of food consumption on the environment can be reduced by switching to a vegetarian diet (no meat consumption).  Please select an appropriate answer.  *Q4*  [1] correct  [2] incorrect  [3] do not know |
| Welche der folgenden Lebensmittel ist mit der tiefsten Klimabelastung (pro 100g) verbunden?  Bitte wählen Sie eine zutreffende Antwort.  *Q5*  [1] Kartoffel-Chips  [2] Brötchen  [3] Salami-Stick  [4] Weiss nicht | Which of the following foods is associated with the lowest climate impact (per 100g)?  Please select an appropriate answer.  *Q5*  [1] potato crisps  [2] bread roll  [3] salami stick  [4] do not know |
| **3.2 Ernährungsbezogenes Umweltwissen in Anlehnung an (Hartmann et al., 2021)** | **3.2 Nutrition-Related Environmental Knowledge Based on (Hartmann et al., 2021)** |
| Welche der folgenden Lebensmittel ist mit der tiefsten Klimabelastung (pro 100g) verbunden?  Bitte wählen Sie eine zutreffende Antwort.  *Q6*  [1] Milchschokolade  [2] Weisse Schokolade  [3] Dunkle Schokolade  [4] Weiss nicht | Which of the following foods is associated with the lowest climate impact (per 100g)?  Please select an appropriate answer.  *Q6*  [1] milk chocolate  [2] white chocolate  [3] dark chocolate  [4] do not know |
| Welche der folgenden Mahlzeiten ist die klimafreundlichste?  Bitte wählen Sie eine zutreffende Antwort.  *Q7*  [1] Pasta mit Rindfleischbolognese  [2] Pasta mit Gemüse-Tomatensauce  [3] Weiss nicht | Which of the following meals is the most climate-friendly?  Please select an appropriate answer.  *Q7*  [1] pasta with beef bolognese  [2] pasta with vegetable-tomato sauce  [3] do not know |
| Welche der folgenden Mahlzeiten ist die klimafreundlichste?  Bitte wählen Sie eine zutreffende Antwort.  *Q8*  [1] Bio-Rindsburger  [2] Bio-Lachsburger  [3] Quinoaburger  [4] Weiss nicht | Which of the following meals is the most climate-friendly?  Please select an appropriate answer.  *Q8*  [1] organic beef burger  [2] organic salmon burger  [3] quinoa burger  [4] do not know |
| Welche der folgenden Mahlzeiten ist die klimafreundlichste?  Bitte wählen Sie eine zutreffende Antwort.  *Q9*  [1] Döner mit Fleisch  [2] Döner mit Falafel  [3] Weiss nicht | Which of the following meals is the most climate-friendly?  Please select an appropriate answer.  *Q9*  [1] kebab with meat  [2] kebab with falafel  [3] do not know |

| Welche der folgenden Mahlzeiten ist die klimafreundlichste?  Bitte wählen Sie eine zutreffende Antwort.  *Q10*  [1] Rindsragout, Kartoffelstock und Bohnen  [2] Geflügelragout, Kartoffelstock und Bohnen  [3] Pilzragout, Kartoffelstock und Bohnen  [4] Weiss nicht | Which of the following meals is the most climate-friendly?  Please select an appropriate answer.  *Q10*  [1] beef stew, mashed potatoes and beans  [2] poultry stew, mashed potatoes and beans  [3] mushroom ragout, mashed potatoes and beans  [4] do not know |
| --- | --- |
| **3.3 Ernährungsbezogenes Umweltwissen in Anlehnung an (Hartmann et al., 2021)** | **3.3 Nutrition-Related Environmental Knowledge Based on (Hartmann et al., 2021)** |
| Welches Produkt ist das umweltfreundlichste?  Bitte wählen Sie eine zutreffende Antwort.  *Q11*  [1] 100 g Spargeln aus Übersee (Flugtransport)  [2] 100 g Schweizer Poulet  [3] 100 g Schweizer Rindfleisch  [4] 100 g Tofu aus Übersee (Schiffstransport)  [5] 100 g Schweizer Schweinefleisch  [6] Weiss nicht | Which product is the most environmentally friendly?  Please select an appropriate answer.  *Q11*  [1] 100 g asparagus from overseas (air transport)  [2] 100 g Swiss chicken  [3] 100 g Swiss beef  [4] 100 g of tofu from overseas (sea transport)  [5] 100 g Swiss pork  [6] do not know |
| Sie möchten ein möglichst umweltfreundliches Abendessen zubereiten. Welche Zutat wählen Sie für die Hauptspeise?  Bitte wählen Sie eine zutreffende Antwort.  *Q12*  [1] Tofu aus Übersee (Schiffstransport)  [2] Schweizer Rinds-Hackbraten  [3] Weiss nicht | You want to prepare a dinner that is as environmentally friendly as possible. Which ingredient do you choose for the main course?  Please select an appropriate answer.  *Q12*  [1] tofu from overseas (transported by ship)  [2] Swiss beef meatloaf  [3] do not know |
| Sie möchten ein möglichst umweltfreundliches Abendessen zubereiten. Welche Beilage wählen Sie?  Bitte wählen Sie eine zutreffende Antwort.  *Q13*  [1] Spargel (Flugimport)  [2] Bohnen (Schiffimport)  [3] Weiss nicht | You want to prepare a dinner that is as environmentally friendly as possible. Which side dish do you choose?  Please select an appropriate answer.  *Q13*  [1] asparagus (imported by air)  [2] beans (imported by ship)  [3] do not know |
| Welches Getränk ist umweltfreundlicher?  Bitte wählen sie eine zutreffende Antwort.  *Q14*  [1] Kaffee  [2] Schwarztee  [3] Weiss nicht | Which beverage is more environmentally friendly?  Please select an appropriate answer.  *Q14*  [1] coffee  [2] black tea  [3] do not know |
| Welches Getränk ist umweltfreundlicher?  Bitte wählen Sie eine zutreffende Antwort.  *Q15*  [1] Mineralwasser  [2] Leitungswasser  [3] Weiss nicht | Which beverage is more environmentally friendly?  Please select an appropriate answer.  *Q15*  [1] mineral water  [2] tap water  [3] do not know |
| Welche der folgenden Früchte können den ganzen Sommer (Juni bis August) in der Schweiz im Freiland geerntet werden? (Mehrere Antworten möglich)  Bitte wählen Sie alle zutreffenden Antworten. | Which of the following fruits can be harvested outdoors in Switzerland throughout the summer (June to August)? (Multiple answers possible)  Please select all applicable answers. |
| Erdbeeren  [0] not quoted  [1] quoted | Strawberries  [0] not quoted  [1] quoted |
| Kirschen  [0] not quoted  [1] quoted | Cherries  [0] not quoted  [1] quoted |
| Birnen  [0] not quoted  [1] quoted | Pears  [0] not quoted  [1] quoted |
| Kiwi  [0] not quoted  [1] quoted | Kiwi  [0] not quoted  [1] quoted |
| Bananen  [0] not quoted  [1] quoted | Bananas  [0] not quoted  [1] quoted |
| Weiss nicht  [0] not quoted  [1] quoted | Do not know  [0] not quoted  [1] quoted |

| **4a. Info** | **4a. Info** |
| --- | --- |
| **4.b Alle_ Messung der Akzeptanz in Anlehnung an (Hagmann et al., 2018)** | **4.b All_ Measurement of acceptance based on (Hagmann et al., 2018)** |
| Geben Sie für die folgenden Strategien an, wie sehr Sie diese unterstützen von 1 (« Finde ich überhaupt nicht gut») bis 7 («Finde ich sehr gut»)  [1] finde ich überhaupt nicht gut  [2]  [3]  [4] neutral  [5]  [6]  [7] finde ich sehr gut | For the following strategies, please indicate how much you support them from 1 (‘I do not support at all’) to 7 (‘I support very much’)  [1] I do not support at all  [2]  [3]  [4] neutral  [5]  [6]  [7] I support very much |
| Informationskampagnen, die über die negativen Umweltauswirkungen von Milchprodukten informieren. | Information campaigns that provide information on the negative environmental impact of dairy products. |
| Stärkeres Gewicht auf fleischlose Rezepte in der Kochschule. | Greater emphasis on meat-free recipes at culinary school. |
| Im Supermarkt werden saisonale Gemüse mit einem Label markiert. | Seasonal vegetables are labelled in the supermarket. |
| Am Verkaufspunkt muss zu allen Lebensmitteln Information zu ihrem Umwelteinfluss (z.B. mit einem Label) gegeben werden. | Information on the environmental impact of all foods (e.g. with a label) must be provided at the point of sale. |
| Im Laden werden Regale mit pflanzlichen Milchalternativen klar markiert (z.B. mit einem grossen grünen Schild). | In the shop, shelves with plant-based milk alternatives are clearly labelled (e.g. with a large green sign). |
| In Kantinen muss das erstgenannte Menu immer fleischlos sein. | In canteens, the initially listed menu must always be meat-free. |
| In Kantinen muss mindestens ein Menu saisonale Gemüse beinhalten. | In canteens, at least one menu must contain seasonal vegetables. |
| In Kantinen werden kleinere Portionen ausgegeben mit der Möglichkeit eines Nachschlags. | In canteens, smaller portions are served with the option of a second helping. |
| Steuer auf Milchprodukte, um den Absatz zu reduzieren. | Tax on dairy products to reduce sales. |
| Steuer auf Fleischprodukte, um den Absatz zu reduzieren. | Tax on meat products to reduce sales. |
| Steuer auf nicht saisonale Gemüse (z.B. Zucchini, Peperoni oder Auberginen im Winter), um den Absatz zu reduzieren. | Tax on vegetables which are out of season (e.g. courgettes, peppers or aubergines in winter) to reduce sales. |
| Steuern auf umweltschädliche Lebensmittel. | Taxes on environmentally harmful food products. |
| Milchalternativen werden subventioniert, damit sie günstiger als Milch sind. | Milk alternatives are subsidised so that they are cheaper than milk. |
| Fleischalternativen werden subventioniert, damit sie günstiger als Fleisch sind. | Meat alternatives are subsidised so that they are cheaper than meat. |
| Saisonales Gemüse (z.B. Kürbis, Lauch, Kabis im Winter) wird subventioniert, um den Absatz zu fördern. | Seasonal vegetables (e.g. pumpkin, leek, cabbage in winter) are subsidised to promote sales. |
| Subventionen auf umweltfreundliche Lebensmittel. | Subsidies on environmentally friendly food. |
| Werbeverbot für Milchprodukte. | Ban on advertisements for dairy products |
| Kantinen müssen an zwei Tagen pro Woche ausschliesslich fleischlose Gerichte anbieten. | Canteens must offer exclusively meat-free dishes two days a week. |
| Im Laden dürfen keine Gemüse angeboten werden, welche per Flugzeug importiert wurden. | In shops, no vegetables may be sold which were imported by plane. |
| **5. Gesundheitsbewusstsein in Anlehung an (Hagmann et al., al., 2018)** | **5. Health Awareness Based on (Hagmann et al., al., 2018)** |
| Bewerten Sie die folgenden Aussagen von 1 ("stimme überhaupt nicht zu") bis 7 ("stimme voll und ganz zu").  [1] stimme überhaupt nicht zu  [2]  [3]  [4] neutral  [5]  [6]  [7] stimme voll und ganz zu | Rate the following statements from 1 (‘strongly disagree’) to 7 (‘fully agree’).  [1] strongly disagree  [2]  [3]  [4] neutral  [5]  [6]  [7] fully agree |
| Mir ist es wichtig, dass ich mich gesund ernähre. | It is important to me that I eat healthily. |
| Meine Gesundheit ist abhängig davon, wie und was ich esse. | My health is dependent on how and what I eat. |
| Wenn man gesund isst, wird man weniger krank. | If you eat healthily, you get sick less often. |
| Ich bin bereit, auf vieles zu verzichten, um möglichst gesund zu essen. | I am prepared to give up a lot in order to eat as healthily as possible. |
| **6. Umweltbewusstsein und -einstellungen (Dunlap et al. 2000 Übersetzung von Alexandra Schleyer-Lindenmann)** | **6. Environmental Awareness and Attitudes (Dunlap et al. 2000 Translation by Alexandra Schleyer-Lindenmann)** |
| Nachfolgend finden Sie einige Aussagen zur Beziehung zwischen Menschen und Umwelt. Geben Sie bitte für jede Aussage an, in welchem Maße Sie mit ihr übereinstimmen von 1 ("Stimme voll und ganz zu") bis 5 ("Stimme überhaupt nicht zu").  [1] stimme voll und ganz zu  [2] stimme zu  [3] stimme weder zu noch lehne ich ab  [4] stimme nicht zu  [5] stimme überhaupt nicht zu | Below you will find some statements on the relationship between humans and the environment. For each statement, please indicate the extent to which you agree with it from 1 (‘I fully agree’) to 5 (‘I strongly disagree’).  [1] I fully agree  [2] I agree  [3] I neither agree nor disagree  [4] I disagree  [5] I strongly disagree |
| Wir nähern uns dem Limit der Bevölkerungszahl, die die Erde bewältigen kann. | We are approaching the limit of the population that the earth can cope with. |
| Die Menschen haben das Recht ihre natürliche Umwelt zu verändern, um sie an ihre Bedürfnisse anzupassen. | People have the right to adapt their natural environment according to their needs. |
| Wenn die Menschen sich in die Natur einmischen, hat das oft katastrophale Konsequenzen. | When people interfere with nature, the consequences are often catastrophic. |
| Der menschliche Einfallsreichtum wird dafür sorgen, dass wir die Erde NICHT unbewohnbar machen. | Human ingenuity will ensure that we do NOT make the earth uninhabitable. |
| Die Menschen missbrauchen die Umwelt schwer. | People abuse the environment severely. |
| **6.2 Umweltbewusstsein und -einstellungen (Dunlap et al. 2000 Übersetzung von Alexandra Schleyer-Lindenmann)** | **6.2 Environmental Awareness and Attitudes (Dunlap et al. 2000 Translation by Alexandra Schleyer-Lindenmann)** |
| Nachfolgend finden Sie einige Aussagen zur Beziehung zwischen Menschen und Umwelt. Geben Sie bitte für jede Aussage an, in welchem Maße Sie mit ihr übereinstimmen von 1 ("Stimme voll und ganz zu") bis 5 ("Stimme überhaupt nicht zu").  [1] stimme voll und ganz zu  [2] stimme zu  [3] stimme weder zu noch lehne ich ab  [4] stimme nicht zu  [5] stimme überhaupt nicht zu | Below you will find some statements on the relationship between humans and the environment. For each statement, please indicate the extent to which you agree with it from 1 (‘I fully agree’) to 5 (‘I strongly disagree’).  [1] I fully agree  [2] I agree  [3] I neither agree nor disagree  [4] I disagree  [5] I strongly disagree |
| Die Erde hat eine Menge natürlicher Ressourcen, wenn wir nur lernen diese zu fördern | The earth has a lot of natural resources, if only we learn to extract them |
| Pflanzen und Tiere haben das gleiche Recht zu leben wie Menschen. | Plants and animals have the same right to live as humans. |
| Das Gleichgewicht der Natur ist stark genug, um den Einfluss der modernen Industrienationen zu bewältigen. | The balance of nature is strong enough to cope with the influence of modern industrialised nations. |
| Abgesehen von unseren speziellen Fähigkeiten sind wir Menschen immer noch den Naturgesetzen unterworfen. | Apart from our special abilities, we humans are still subject to the laws of nature. |
| Die sogenannte “Umweltkrise” mit der die Menschheit konfrontiert ist, wird völlig übertrieben. | The so-called ‘environmental crisis’ that humanity is facing is completely exaggerated. |

| **6.3 Umweltbewusstsein und -einstellungen (Dunlap et al. 2000 Übersetzung von Alexandra Schleyer-Lindenmann)** | **6.3 Environmental Awareness and Attitudes (Dunlap et al. 2000 Translation by Alexandra Schleyer-Lindenmann)** |
| --- | --- |
| Nachfolgend finden Sie einige Aussagen zur Beziehung zwischen Menschen und Umwelt. Geben Sie bitte für jede Aussage an, in welchem Maße Sie mit ihr übereinstimmen von 1 ("Stimme voll und ganz zu") bis 5 ("Stimme überhaupt nicht zu").  [1] stimme voll und ganz zu  [2] stimme zu  [3] stimme weder zu noch lehne ich ab  [4] stimme nicht zu  [5] stimme überhaupt nicht zu | Below you will find some statements on the relationship between humans and the environment. For each statement, please indicate the extent to which you agree with it from 1 (‘I fully agree’) to 5 (‘I strongly disagree’).  [1] I fully agree  [2] I agree  [3] I neither agree nor disagree  [4] I disagree  [5] I strongly disagree |
| Die Erde ist wie ein Raumschiff mit wenig Platz und wenig Ressourcen. | Earth is like a spaceship with little space and few resources. |
| Menschen sind dazu geboren, über den Rest der Natur zu herrschen. | Humans are born to rule over the rest of nature. |
| Das Gleichgewicht der Natur ist sehr empfindlich und leicht zu stören. | The balance of nature is very delicate and easily disturbed. |
| Die Menschen werden möglicherweise genug darüber lernen, wie die Naturfunktioniert, um sie kontrollieren zu können. | People may learn enough about how nature works to be able to control it. |
| Wenn die Dinge so weitergehen wie bisher, werden wir bald eine große Umweltkatastrophe erleben. | If things continue as they are, we will soon experience a major environmental catastrophe. |
| **7. Verantwortungsbewusstsein** | **7. Sense of Responsibility** |
| Bewerten Sie die folgenden Akteure. Wieviel Verantwortung tragen die einzelnen Akteure für nachhaltigen Konsum von Lebensmitteln? 1 ("Gar keine Verantwortung ") bis 7 (" Sehr viel Verantwortung").  [1] gar keine Verantwortung  [2]  [3]  [4] neutral  [5]  [6]  [7] sehr viel Verantwortung | Evaluate the following players. How much responsibility do the individual actors take for sustainable food consumption? 1 (‘no responsibility at all’) to 7 (‘a great amount of responsibility’).  [1] no responsibility at all  [2]  [3]  [4] neutral  [5]  [6]  [7] a great amount of responsibility |
| Produktion/ Landwirtschaft | Production/ Agriculture |
| Handel (z.B. über das Angebot beim Detailhändler) | Retail (e.g. regarding the offer at retailers) |
| Konsumenten/innen (z.B. über den Konsumverhalten) | Consumers (e.g. regarding consumer behaviour) |
| Staat/Politik (z.B. über Gesetze, Subventionen) | Government/Politics (e.g. regarding laws, subsidies) |
| **7.2 Verantwortungsbewusstsein** | **7.2 Sense of Responsibility** |
| Wie zuversichtlich sind Sie, dass nachfolgende Akteure den nachhaltigen Konsum von Lebensmitteln fördern? 1 ("Gar keine Zuversichtlichkeit ") bis 7 (" Sehr viel Zuversichtlichkeit").  [1] gar keine Zuversicht  [2]  [3]  [4] neutral  [5]  [6]  [7] sehr viel Zuversicht | How confident are you that the following players will promote the sustainable consumption of food? 1 (‘not at all confident’) to 7 (‘very confident’).  [1] no at all confident  [2]  [3]  [4] neutral  [5]  [6]  [7] very confident |
| Produktion/ Landwirtschaft | Production/ Agriculture |
| Handel (z.B. über das Angebot beim Detailhändler) | Retail (e.g. regarding the offer at retailers) |
| Konsumenten/innen (z.B. über den Konsumverhalten) | Consumers (e.g. regarding consumer behaviour) |
| Staat/Politik (z.B. über Gesetze, Subventionen) | Government/Politics (e.g. regarding laws, subsidies) |
| **Abschluss** | **Conclusion** |
| Falls Sie noch Fragen oder Anregungen haben, können Sie diese hier notieren. Ansonsten klicken Sie bitte auf "weiter".  [Kommentar] | If you have any further questions or suggestions, please feel free to note them here. Otherwise, please click on ‘next’.  [Comment] |
